# On the Use of Chlorate of Potash in Mercurial Stomatitis

**Published:** 1856-02

**Authors:** 


*On the Use of Chlorate of Potash in Mercurial Stomatitis.*—From the experiments of M. Herpin, of Geneva, from those of M. Blache (*Gazette Hebdomadaire*, vol. ii., No. 8, p. 147), as well as from some well-detailed facts that M. Demarqua has just reported, it would appear that chlorate of potash, given internally, arrests, with rapidity and certainty, the effects of mercurial stomatitis. This effect has been established in patients in whom the mercurial intoxication supervened on the exhibition of mercury for syphilis, puerperal peritonitis, and ophthalmia.

The chlorate is administered in a mucilaginous mixture, the dose commencing with half a drachm, which is frequently sufficient to remove the symptoms. But it has been given to the extent of four scruples, two and a half drachms, half an ounce, and upwards.

As this medicine, notwithstanding its remarkable efficacy, is by no means a specific, we must not neglect to combine with it local astringents and caustics, which, even alone, possess so powerful an action in mercurial ptyalism.

M. Gustin, intern in pharmacy, wishing, for the sake of experiment, to submit himself to the action of chlorate of patash, took two drachms at nine o'clock in the evening. On awaking, a sort of astriction, with slight nausea, was perceived in the mouth; the gums were a little rough to the touch. Although the saliva was not sensibly lessened, it appeared to him to be more watery than usual. This observer has also proved that the chlorate of potash is, in great part, eliminated by the urinary secretion.—*Bulletin Général de Thérapeutique* and *Gazette Hebdomadaire. N. Y. Journal of Medicine.*

